# Benign Metastasizing Leiomyoma: A Rare Type of Lung Metastases—Two Case Reports and Review of the Literature

**DOI:** 10.1155/2014/842801

**Published:** 2014-02-12

**Authors:** Rokana Taftaf, Sandra Starnes, Jiang Wang, Ralph Shipley, Tariq Namad, Rana Khaled, Nagla Abdel Karim

**Affiliations:** ^1^Department of Medicine, Division of Hematology/Oncology, University of Cincinnati, Cincinnati, OH 45219, USA; ^2^Department of Surgery, Division of Thoracic Surgery, University of Cincinnati, Cincinnati, OH 45219, USA; ^3^Department of Pathology, University of Cincinnati, Cincinnati, OH 45219, USA; ^4^Department of Radiology, University of Cincinnati, Cincinnati, OH 45219, USA

## Abstract

Benign metastasizing leiomyoma (BML) is a rare disease that usually occurs in women of reproductive age. They typically have history of uterine leiomyoma treated with hysterectomy. BML can metastasize to distant organs, with the lung being the most common organ. We report two patients who presented with benign metastasizing leiomyoma to the lung. Our first case was a fifty-two-year-old female who presented with multiple lung masses, with a past medical history of uterine leiomyoma who underwent hysterectomy 17 years ago. A CT-guided biopsy showed benign appearing spindle cells and pathology confirmed her diagnosis with additional positive estrogen/progesterone receptor stains. Our second case was a fifty-six-year-old female who presented with multiple cavitary pulmonary nodules. She subsequently underwent a video-assisted thoracoscopic surgery (VATS) with wedge resection of one of the nodules. Pathology confirmed the diagnosis based on morphology and immunohistochemical staining strongly positive for estrogen/progesterone receptors. Benign metastasizing leiomyoma is a rare condition which may affect women of reproductive age. This should be considered in the differential in patients who present with multiple pulmonary nodules, especially with a history of uterine leiomyoma. Additional stains, such as estrogen/progesterone receptors, may need to be done to confirm the diagnosis.

## 1. Introduction

Benign metastasizing leiomyoma (BML) represents a rare disease entity. This may present as lesions in lymph nodes, deep soft tissues, mesentery, bones, central nervous system, and heart. However, the most commonly affected organ is the lung; thus, it might be confused with leiomyosarcoma [1]. BMLs occur most commonly in women during their reproductive years. The preponderance of diagnosis happens in patients who develop new pulmonary nodules many years after the removal of the uterus for leiomyoma [2–11].

Here we report two patients who presented with the unusual presentation of benign metastasizing leiomyomas to the lungs.

## 2. Report of Two Cases

### 2.1. First Case

**Figure 1:**
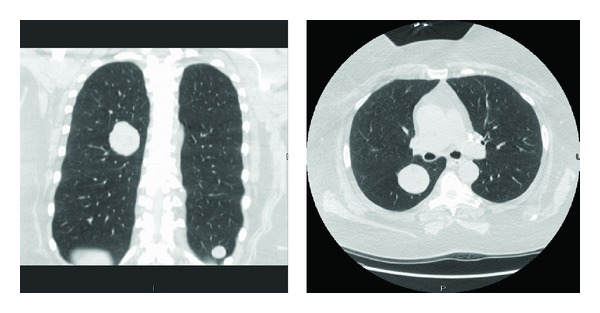
CT: well-defined noncalcified pulmonary nodules.

A fifty-two-year-old female presented with abdominal pain. An abdominal computed tomography (CT) scan demonstrated a right lower lobe mass. She then underwent a chest CT which showed a right lower lobe (RLL) mass (3.9 × 3.5 cm) with numerous smaller nodules throughout both lungs (CT, Figure 1). A positron emission tomography (PET) scan showed positive uptake in the larger right lower lobe nodule and a left lower lobe nodule. Her medical history included a hysterectomy for a benign leiomyoma 17 years ago. On review of systems she reported cough, shortness of breath, and diarrhea but denied any constitutional symptoms or complaints in other systems. She was a current smoker with a history of smoking 1 pack per day for the last 34 years. On physical examination she was found to have decreased breath sounds in her right middle and lower fields. No other abnormality was appreciated.

**Figure 2:**
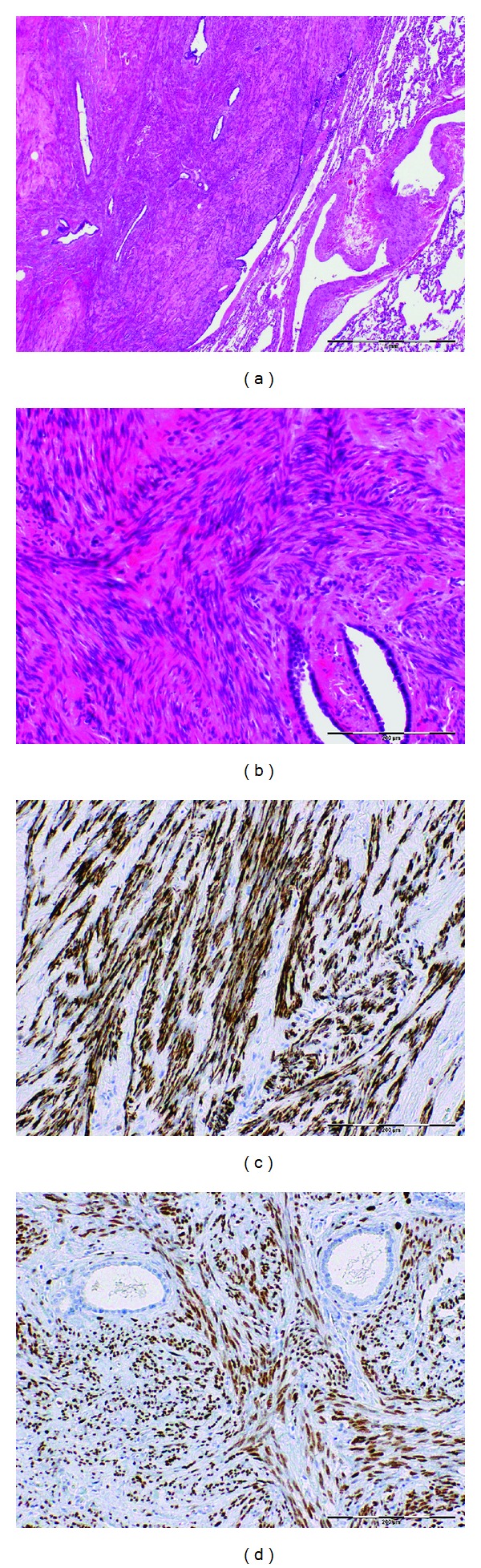
(a) and (b) H&E: broad fascicles of bland spindle cells with entrapped scattered tubules lined by bronchoepithelial cells. (c) and (d) Immunohistochemistry, tumor cells are strongly positive for muscular marker desmin (c) and ER (d).

She underwent a bronchoscopy and endobronchial ultrasound (EBUS) with biopsy of the right lower lobe (RLL) lung nodule and a 12R lymph node. These biopsies were nondiagnostic, so she underwent a CT-guided fine needle aspiration which showed benign appearing spindle cells. Given the persistent concern for malignancy, she was referred to our institution for further evaluation. Consequently, she underwent a video-assisted thoracoscopic surgery (VATS) with wedge resection of the left lower lobe lesion. Pathology microscopic examination demonstrated well-demarcated nodules composed of bland spindle cells forming broad fascicles. There were entrapped scattered mature adipose tissue and tubules lined by benign bronchoepithelial cells (Figures 2(a) and 2(b)). No mitosis or necrosis was identified. Initial interpretation was leiomyomatous hamartoma with no evidence of malignancy or atypia. However, given the fact that she had a hysterectomy for uterine leiomyoma, the lesion was stained for estrogen/progesterone receptor by immunohistochemistry (Figure 2(d)). The final diagnosis was benign metastasizing leiomyoma (BML).

### 2.2. Second Case

A fifty-six-year-old female presented to us with recently diagnosed multiple pulmonary cavitary nodules. Her past medical history included coronary artery disease, previous myocardial infarction, hyperlipidemia, diabetes type 2, Grave's disease, and a right renal stent for renal artery stenosis. She has no prior history of malignancy. On review of systems, she reported a dry cough, chest pain, dyspnea on exertion, palpitations, fatigue, weight gain, urinary frequency, and nocturia. She smoked 1 pack per day for 15 years and quit smoking 2 years prior. Her physical exam, including lung exam, was unremarkable.

**Figure 3:**
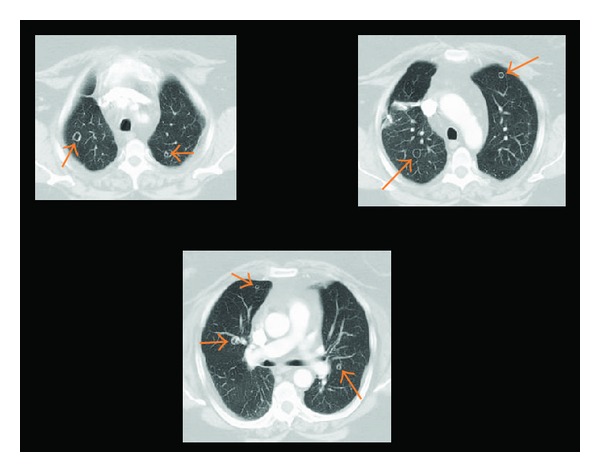
CT: multiple bilateral cavitary pulmonary nodules.

On chest CT angiography imaging, multiple bilateral, relatively thick-walled cavitary pulmonary nodules were found, measuring approximately 1 cm in size with prevalence in the upper lobes (Figure 3). She underwent a VATS wedge resection of the right upper lobe nodules. On microscopy, there were subpleural well-defined nodules comprised of bland spindle to oval cells with scant cytoplasm arranged in bundles and fascicles. The spindle cells appeared to be smooth muscle in nature which was supported by diffuse positive immunohistochemical staining for desmin, smooth muscle actin, caldesmon, and vimentin. They were also diffusely and strongly positive for estrogen and progesterone receptors and negative for HMB-45. Final pathological interpretation was benign metastasizing leiomyoma based on morphology and immunohistochemical staining.

## 3. Discussion

Benign metastasizing leiomyoma (BML) is a rare disease that usually occurs in women of reproductive age with a history of uterine leiomyoma treated with hysterectomy. BML can metastasize to distant organs, such as the lung, skin, bone, mediastinum, lymph nodes, muscular tissue, heart, and retroperitoneum [12]. Most patients with BML are diagnosed incidentally as they usually are symptoms-free [5]. If present, symptoms typically include cough, shortness of breath, and chest pain. BML lesions tend to appear several years after the diagnosis of the uterine leiomyomas. In our two cases, the interval was 17 years for the first case and new onset for the second case. In a report of ten cases of BML, this interval ranged from 4 to 23 years with a mean of 14.9 years [6]. In literature review by Jautzke et al., the authors analyzed 74 reported cases of BML [5]. The interval between hysterectomy and the diagnosis of BML ranged from 3 to 20 years (mean 10 years).

Although the specific etiology of BML has not been fully established, the presence of hormone receptors (estrogen/progesterone receptors) and amenability to antihormonal treatments strongly suggest a uterine origin [6]. However, these tumors should be well distinguished from the true malignant sarcomas. The most helpful pathologic features that characterize BML are the low mitotic index and the absence of coagulative necrosis and atypia [13].

Since these tumors are rare, no definite treatment strategy has been established. Treatments used include surgical removal of the lung lesions. For multiple lesions not amenable to removeal, conservative antiestrogen management has been tried using oopherectomy or gonadotropin-releasing hormone analogue. Other antiestrogen therapies such as selective estrogen receptor modulator, progestrone, and aromotase inhibitor have been suggested [9, 12].

With regard to the prognosis, patients with BML tend to have a favorable clinical course and outcome. Out of ten patients reported in a case series [6], only one died of BML complications. Median survival for the ten cases was 94 months with a range between 7 and 101 months.

## 4. Conclusion

Benign metastasizing leiomyoma should be in the differential diagnosis in females of reproductive age who present with pulmonary nodules and have a history of uterine leiomyoma. Other types of spindle cell neoplasm, such as sarcoma, nerve sheath tumor, and malignant melanoma need to be excluded. Immunostains to demonstrate smooth muscle origin and positivity of estrogen and progesterone receptor may be helpful in establishing the diagnosis.
